# 1,2-Di-2-pyridylethyl­ene–phenyl­succinic acid (1/1)

**DOI:** 10.1107/S1600536808040245

**Published:** 2008-12-20

**Authors:** Qun-Zeng Huang, Heng-Zhen Shi

**Affiliations:** a College of Chemistry and Pharmacy Engineering, Nanyang Normal University, Nanyang 473061, People’s Republic of China

## Abstract

In the title 1:1 adduct, C_10_H_10_O_4_·C_12_H_10_N_2_, the two components are linked by O—H⋯N hydrogen bonds to form a one-dimensional chain. The dihedral angle between the pyridine rings is 15.68 (8)° These chains are further inter­connected by weak inter­molecular C—H⋯O hydrogen bonds and weak C—H⋯π inter­actions to generate a three-dimensional network.

## Related literature

For (*S*)- and (*R,S*)-phenyl­succinic acids, see: Fischer & Profir (2003*a* ▶,*b* ▶). For weak C—H⋯O hydrogen bonds, see, for example: Bhogala *et al.* (2005 ▶); Wang *et al.* (2008 ▶). For C—H⋯π inter­actions, see, for example: Fun & Kia (2008 ▶). 
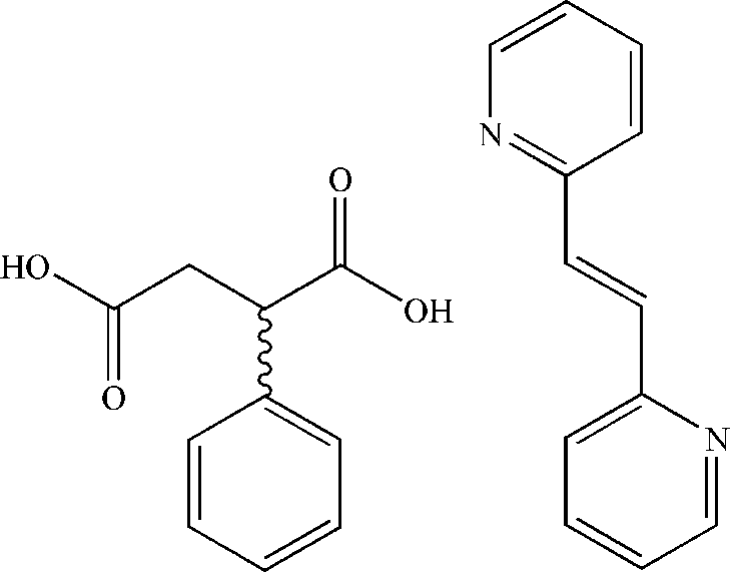


## Experimental

### 

#### Crystal data

C_10_H_10_O_4_·C_12_H_10_N_2_
*M* *_r_* = 376.40
Triclinic, 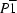

*a* = 8.6707 (13) Å
*b* = 10.9714 (17) Å
*c* = 11.2013 (17) Å
α = 80.801 (2)°
β = 69.696 (2)°
γ = 77.663 (2)°
*V* = 972.0 (3) Å^3^
*Z* = 2
Mo *K*α radiation
μ = 0.09 mm^−1^
*T* = 291 (2) K
0.34 × 0.33 × 0.29 mm

#### Data collection

Bruker SMART CCD area-detector diffractometer
Absorption correction: multi-scan (*SADABS*; Bruker, 1997 ▶) *T*_min_ = 0.957, *T*_max_ = 0.975
7261 measured reflections
3605 independent reflections
2369 reflections with *I* > 2σ(*I*)
*R*_int_ = 0.022

#### Refinement

*R*[*F* ^2^ > 2σ(*F* ^2^)] = 0.047
*wR*(*F* ^2^) = 0.133
*S* = 1.03
3605 reflections
253 parameters
H-atom parameters constrained
Δρ_max_ = 0.19 e Å^−3^
Δρ_min_ = −0.20 e Å^−3^

### 

Data collection: *SMART* (Bruker, 1997 ▶); cell refinement: *SAINT* (Bruker, 1997 ▶); data reduction: *SAINT*; program(s) used to solve structure: *SHELXS97* (Sheldrick, 2008 ▶); program(s) used to refine structure: *SHELXL97* (Sheldrick, 2008 ▶); molecular graphics: *SHELXTL* (Sheldrick, 2008 ▶); software used to prepare material for publication: *SHELXTL*.

## Supplementary Material

Crystal structure: contains datablocks I, global. DOI: 10.1107/S1600536808040245/si2134sup1.cif

Structure factors: contains datablocks I. DOI: 10.1107/S1600536808040245/si2134Isup2.hkl

Additional supplementary materials:  crystallographic information; 3D view; checkCIF report

## Figures and Tables

**Table 1:** Hydrogen-bond geometry (Å, °)

*D*—H⋯*A*	*D*—H	H⋯*A*	*D*⋯*A*	*D*—H⋯*A*
O4—H4⋯N1	0.82	1.88	2.699 (2)	176
O1—H1⋯N2^i^	0.82	1.88	2.683 (2)	165
C2—H2⋯O2^ii^	0.93	2.56	3.472 (3)	164
C11—H11⋯O3^iii^	0.93	2.43	3.350 (3)	170
C3—H3⋯*Cg*1^iv^	0.93	2.86	3.650 (4)	143

Symmetry codes: (i) 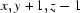; (ii) 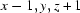; (iii) 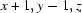; (iv) 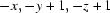. *Cg*1 is the centroid of the C15–C20 phenyl ring.

**Fig. 1.**
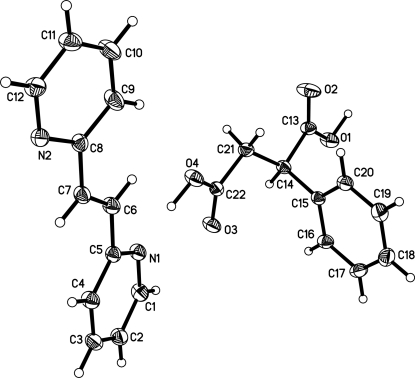
A view of the heteromolecular components of the title compound with the atom labelling scheme. Displacement ellipsoids are drawn at the 30% probability level.

**Fig. 2.**
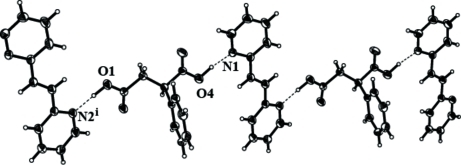
A view of the one-dimensional hydrogen-bonding pattern network.

**Fig. 3.**
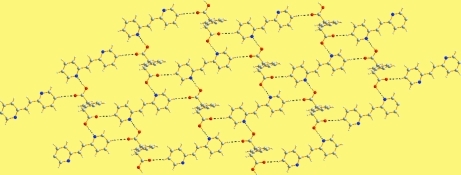
The crystal packing of (I), showing the two-dimensional structure.

**Fig. 4.**
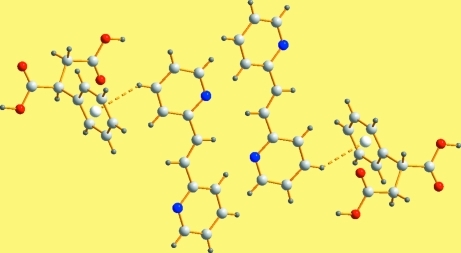
A view of the C—H···π interactions in the crystal structure of the title compound.

## supplementary crystallographic information

### Comment

The asymmetric unit consists of the two heteromolecular components of the title compound in a (1/1) ratio (Fig.1). These molecules are linked by intermolecular O—H···N hydrogen bonds to form a one-dimensional chain (Table 1, Fig.2). The chains interact with each other *via* weak intermolecular C—H···O hydrogen bonds and C—H···π interactions (Tab 1). *Cg*1 is the centroid of the phenyl ring C15 - C20. These hydrogen bonds, albeit rather weak, link the chains into two-dimensional layers structure (Fig. 3), which are further connected by weak intermolecular C—H···π interactions to generate a three-dimensional supramolecular structure (Fig. 4).

### Experimental

1,2-bis(2-pyridyl)ethylene (0.15 mmol), (*R*)-Phenylsuccinic acid (0.15 mmol), and NaOH (0.1 mmol) were added to a H_2_O solution (20 ml) in a Teflonlined stainless steel reactor. The mixture was heated at 450 K for 4 d, and then slowly cooled down to room temperature. Colorless crystals of the title compound were obtained.

### Refinement

All H atoms were positioned geometrically and refined as riding atoms, with C—H = 0.93 Å (aromatic H) and *U*ĩso~(H) = 1.2Ueq(C), with C—H = 0.97 Å (CH2) and *U*ĩso~(H) = 1.2Ueq(C), with C—H = 0.98 Å(CH) and *U*ĩso~(H) = 1.2Ueq(C) and with O—H = 0.82 Å (OH) and *U*ĩso~(H) = 1.5Ueq(O).

### Crystal data

**Table:**

C_10_H_10_O_4_·C_12_H_10_N_2_	*Z* = 2
*M**_r_* = 376.40	*F*(000) = 396
Triclinic, *P*1	*D*_x_ = 1.286 Mg m^−^^3^
Hall symbol: -P 1	Mo *K*α radiation, λ = 0.71073 Å
*a* = 8.6707 (13) Å	Cell parameters from 1614 reflections
*b* = 10.9714 (17) Å	θ = 2.5–26.0°
*c* = 11.2013 (17) Å	µ = 0.09 mm^−^^1^
α = 80.801 (2)°	*T* = 291 K
β = 69.696 (2)°	Block, colorless
γ = 77.663 (2)°	0.34 × 0.33 × 0.29 mm
*V* = 972.0 (3) Å^3^	

### Data collection

**Table:**

Bruker SMART CCD area-detector diffractometer	3605 independent reflections
Radiation source: fine-focus sealed tube	2369 reflections with *I* > 2σ(*I*)
graphite	*R*_int_ = 0.022
φ and ω scans	θ_max_ = 25.5°, θ_min_ = 2.5°
Absorption correction: multi-scan (*SADABS*; Bruker, 1997)	*h* = −10→10
*T*_min_ = 0.957, *T*_max_ = 0.975	*k* = −13→13
7261 measured reflections	*l* = −13→13

### Refinement

**Table:**

Refinement on *F*^2^	Primary atom site location: structure-invariant direct methods
Least-squares matrix: full	Secondary atom site location: difference Fourier map
*R*[*F*^2^ > 2σ(*F*^2^)] = 0.047	Hydrogen site location: inferred from neighbouring sites
*wR*(*F*^2^) = 0.133	H-atom parameters constrained
*S* = 1.03	*w* = 1/[σ^2^(*F*_o_^2^) + (0.0553*P*)^2^ + 0.1924*P*] where *P* = (*F*_o_^2^ + 2*F*_c_^2^)/3
3605 reflections	(Δ/σ)_max_ < 0.001
253 parameters	Δρ_max_ = 0.19 e Å^−^^3^
0 restraints	Δρ_min_ = −0.19 e Å^−^^3^

### Fractional atomic coordinates and isotropic or equivalent isotropic displacement parameters (Å^2^)

**Table:**

	*x*	*y*	*z*	*U*_iso_*/*U*_eq_	
O1	0.47679 (18)	0.96437 (13)	−0.17709 (14)	0.0637 (5)	
H1	0.5453	0.9815	−0.2464	0.096*	
O2	0.6712 (2)	0.79683 (17)	−0.17226 (16)	0.0896 (7)	
O3	0.27415 (18)	0.75636 (14)	0.27577 (13)	0.0620 (4)	
O4	0.45071 (18)	0.57475 (13)	0.26650 (13)	0.0618 (4)	
H4	0.3867	0.5601	0.3387	0.093*	
N1	0.2501 (2)	0.51408 (15)	0.50443 (15)	0.0485 (4)	
N2	0.66326 (19)	0.01005 (14)	0.57849 (15)	0.0454 (4)	
C1	0.1106 (3)	0.5966 (2)	0.5536 (2)	0.0546 (6)	
H1A	0.0995	0.6764	0.5110	0.065*	
C2	−0.0168 (3)	0.5695 (2)	0.6635 (2)	0.0567 (6)	
H2	−0.1108	0.6295	0.6947	0.068*	
C3	−0.0007 (3)	0.4507 (2)	0.7260 (2)	0.0561 (6)	
H3	−0.0845	0.4289	0.7999	0.067*	
C4	0.1410 (3)	0.3649 (2)	0.6774 (2)	0.0531 (5)	
H4A	0.1531	0.2846	0.7187	0.064*	
C5	0.2663 (2)	0.39814 (18)	0.56644 (18)	0.0434 (5)	
C6	0.4216 (3)	0.31193 (19)	0.5089 (2)	0.0503 (5)	
H6	0.4894	0.3386	0.4284	0.060*	
C7	0.4745 (2)	0.20028 (18)	0.56004 (19)	0.0470 (5)	
H7	0.4071	0.1742	0.6409	0.056*	
C8	0.6279 (2)	0.11358 (18)	0.50279 (18)	0.0440 (5)	
C9	0.7339 (3)	0.1334 (2)	0.3775 (2)	0.0657 (7)	
H9	0.7090	0.2046	0.3256	0.079*	
C10	0.8761 (3)	0.0464 (2)	0.3313 (2)	0.0720 (7)	
H10	0.9480	0.0595	0.2485	0.086*	
C11	0.9108 (3)	−0.0587 (2)	0.4075 (2)	0.0624 (6)	
H11	1.0050	−0.1187	0.3775	0.075*	
C12	0.8017 (3)	−0.07322 (19)	0.5304 (2)	0.0549 (6)	
H12	0.8252	−0.1445	0.5827	0.066*	
C13	0.5369 (2)	0.85814 (19)	−0.12306 (19)	0.0462 (5)	
C14	0.4165 (2)	0.81888 (18)	0.00741 (18)	0.0427 (5)	
H14	0.3682	0.8931	0.0550	0.051*	
C15	0.2750 (2)	0.77139 (17)	−0.01329 (17)	0.0399 (5)	
C16	0.1120 (2)	0.83595 (19)	0.02700 (19)	0.0488 (5)	
H16	0.0881	0.9074	0.0694	0.059*	
C17	−0.0154 (3)	0.7948 (2)	0.0046 (2)	0.0588 (6)	
H17	−0.1236	0.8394	0.0316	0.071*	
C18	0.0169 (3)	0.6883 (2)	−0.0573 (2)	0.0635 (6)	
H18	−0.0688	0.6612	−0.0723	0.076*	
C19	0.1789 (3)	0.6220 (2)	−0.0970 (2)	0.0603 (6)	
H19	0.2018	0.5498	−0.1381	0.072*	
C20	0.3064 (3)	0.66335 (19)	−0.07542 (19)	0.0497 (5)	
H20	0.4145	0.6186	−0.1026	0.060*	
C21	0.5086 (2)	0.7212 (2)	0.08432 (18)	0.0501 (5)	
H21A	0.5541	0.6462	0.0396	0.060*	
H21B	0.6013	0.7538	0.0899	0.060*	
C22	0.3968 (2)	0.68718 (19)	0.21791 (18)	0.0438 (5)	

### Atomic displacement parameters (Å^2^)

**Table:**

	*U*^11^	*U*^22^	*U*^33^	*U*^12^	*U*^13^	*U*^23^
O1	0.0595 (9)	0.0537 (9)	0.0538 (9)	−0.0004 (7)	−0.0032 (7)	0.0158 (7)
O2	0.0579 (10)	0.0906 (13)	0.0669 (11)	0.0189 (9)	0.0120 (9)	0.0282 (9)
O3	0.0591 (9)	0.0598 (10)	0.0442 (8)	0.0097 (8)	−0.0023 (7)	0.0005 (7)
O4	0.0653 (10)	0.0513 (9)	0.0446 (8)	0.0079 (7)	−0.0029 (7)	0.0062 (7)
N1	0.0520 (10)	0.0422 (10)	0.0428 (9)	−0.0012 (8)	−0.0126 (8)	0.0046 (8)
N2	0.0471 (9)	0.0384 (9)	0.0450 (9)	−0.0050 (7)	−0.0134 (8)	0.0056 (7)
C1	0.0621 (14)	0.0425 (12)	0.0498 (13)	0.0039 (10)	−0.0165 (11)	0.0013 (10)
C2	0.0534 (13)	0.0584 (14)	0.0490 (13)	0.0073 (11)	−0.0135 (10)	−0.0080 (11)
C3	0.0525 (13)	0.0640 (15)	0.0420 (12)	−0.0079 (11)	−0.0068 (10)	0.0006 (11)
C4	0.0551 (13)	0.0464 (12)	0.0479 (12)	−0.0053 (10)	−0.0107 (10)	0.0051 (10)
C5	0.0458 (11)	0.0419 (11)	0.0398 (11)	−0.0055 (9)	−0.0141 (9)	0.0012 (9)
C6	0.0508 (12)	0.0475 (12)	0.0425 (11)	−0.0031 (10)	−0.0098 (9)	0.0052 (9)
C7	0.0514 (12)	0.0457 (12)	0.0387 (11)	−0.0053 (9)	−0.0122 (9)	0.0011 (9)
C8	0.0495 (11)	0.0413 (11)	0.0397 (11)	−0.0062 (9)	−0.0150 (9)	−0.0005 (9)
C9	0.0788 (16)	0.0581 (15)	0.0403 (12)	0.0049 (12)	−0.0092 (11)	0.0063 (11)
C10	0.0762 (17)	0.0708 (17)	0.0427 (13)	0.0047 (13)	0.0013 (12)	−0.0010 (12)
C11	0.0553 (13)	0.0549 (14)	0.0608 (14)	0.0042 (11)	−0.0055 (11)	−0.0083 (12)
C12	0.0521 (12)	0.0431 (12)	0.0600 (14)	−0.0005 (10)	−0.0148 (11)	0.0038 (10)
C13	0.0405 (11)	0.0446 (12)	0.0451 (11)	−0.0064 (9)	−0.0083 (9)	0.0060 (9)
C14	0.0421 (10)	0.0409 (11)	0.0375 (10)	−0.0041 (8)	−0.0072 (8)	0.0005 (8)
C15	0.0393 (10)	0.0417 (11)	0.0325 (10)	−0.0047 (8)	−0.0081 (8)	0.0034 (8)
C16	0.0436 (11)	0.0463 (12)	0.0477 (12)	−0.0027 (9)	−0.0091 (9)	0.0012 (9)
C17	0.0378 (11)	0.0715 (16)	0.0586 (14)	−0.0083 (11)	−0.0134 (10)	0.0115 (12)
C18	0.0639 (15)	0.0765 (17)	0.0579 (14)	−0.0299 (13)	−0.0258 (12)	0.0094 (13)
C19	0.0706 (16)	0.0599 (14)	0.0529 (13)	−0.0190 (12)	−0.0183 (12)	−0.0052 (11)
C20	0.0473 (12)	0.0501 (13)	0.0457 (12)	−0.0033 (10)	−0.0113 (9)	−0.0025 (10)
C21	0.0409 (11)	0.0568 (13)	0.0450 (12)	−0.0067 (9)	−0.0094 (9)	0.0039 (10)
C22	0.0433 (11)	0.0477 (12)	0.0370 (10)	−0.0047 (9)	−0.0117 (9)	−0.0018 (9)

### Geometric parameters (Å, °)

**Table:**

O1—C13	1.315 (2)	C9—H9	0.9300
O1—H1	0.8200	C10—C11	1.365 (3)
O2—C13	1.204 (2)	C10—H10	0.9300
O3—C22	1.209 (2)	C11—C12	1.380 (3)
O4—C22	1.324 (2)	C11—H11	0.9300
O4—H4	0.8200	C12—H12	0.9300
N1—C1	1.347 (2)	C13—C14	1.531 (3)
N1—C5	1.352 (2)	C14—C15	1.529 (3)
N2—C12	1.344 (2)	C14—C21	1.532 (3)
N2—C8	1.352 (2)	C14—H14	0.9800
C1—C2	1.380 (3)	C15—C16	1.391 (3)
C1—H1A	0.9300	C15—C20	1.399 (3)
C2—C3	1.381 (3)	C16—C17	1.389 (3)
C2—H2	0.9300	C16—H16	0.9300
C3—C4	1.378 (3)	C17—C18	1.383 (3)
C3—H3	0.9300	C17—H17	0.9300
C4—C5	1.395 (3)	C18—C19	1.390 (3)
C4—H4A	0.9300	C18—H18	0.9300
C5—C6	1.474 (3)	C19—C20	1.387 (3)
C6—C7	1.326 (3)	C19—H19	0.9300
C6—H6	0.9300	C20—H20	0.9300
C7—C8	1.465 (3)	C21—C22	1.513 (3)
C7—H7	0.9300	C21—H21A	0.9700
C8—C9	1.398 (3)	C21—H21B	0.9700
C9—C10	1.382 (3)		
			
C13—O1—H1	109.5	N2—C12—H12	118.2
C22—O4—H4	109.5	C11—C12—H12	118.2
C1—N1—C5	118.17 (17)	O2—C13—O1	123.53 (18)
C12—N2—C8	118.53 (17)	O2—C13—C14	123.50 (18)
N1—C1—C2	123.70 (19)	O1—C13—C14	112.95 (16)
N1—C1—H1A	118.1	C15—C14—C13	108.73 (16)
C2—C1—H1A	118.1	C15—C14—C21	112.41 (16)
C1—C2—C3	118.01 (19)	C13—C14—C21	110.86 (15)
C1—C2—H2	121.0	C15—C14—H14	108.2
C3—C2—H2	121.0	C13—C14—H14	108.2
C4—C3—C2	119.24 (19)	C21—C14—H14	108.2
C4—C3—H3	120.4	C16—C15—C20	118.20 (19)
C2—C3—H3	120.4	C16—C15—C14	120.81 (18)
C3—C4—C5	120.13 (19)	C20—C15—C14	120.98 (17)
C3—C4—H4A	119.9	C17—C16—C15	120.7 (2)
C5—C4—H4A	119.9	C17—C16—H16	119.6
N1—C5—C4	120.74 (17)	C15—C16—H16	119.6
N1—C5—C6	115.87 (17)	C18—C17—C16	120.7 (2)
C4—C5—C6	123.39 (18)	C18—C17—H17	119.7
C7—C6—C5	126.66 (19)	C16—C17—H17	119.7
C7—C6—H6	116.7	C17—C18—C19	119.3 (2)
C5—C6—H6	116.7	C17—C18—H18	120.4
C6—C7—C8	126.93 (19)	C19—C18—H18	120.4
C6—C7—H7	116.5	C20—C19—C18	120.1 (2)
C8—C7—H7	116.5	C20—C19—H19	120.0
N2—C8—C9	120.45 (18)	C18—C19—H19	120.0
N2—C8—C7	116.06 (17)	C19—C20—C15	121.01 (19)
C9—C8—C7	123.49 (18)	C19—C20—H20	119.5
C10—C9—C8	119.6 (2)	C15—C20—H20	119.5
C10—C9—H9	120.2	C22—C21—C14	112.58 (16)
C8—C9—H9	120.2	C22—C21—H21A	109.1
C11—C10—C9	119.9 (2)	C14—C21—H21A	109.1
C11—C10—H10	120.0	C22—C21—H21B	109.1
C9—C10—H10	120.0	C14—C21—H21B	109.1
C10—C11—C12	118.0 (2)	H21A—C21—H21B	107.8
C10—C11—H11	121.0	O3—C22—O4	123.46 (18)
C12—C11—H11	121.0	O3—C22—C21	123.82 (18)
N2—C12—C11	123.6 (2)	O4—C22—C21	112.68 (16)
			
C5—N1—C1—C2	−0.1 (3)	O2—C13—C14—C15	−104.5 (2)
N1—C1—C2—C3	−0.5 (3)	O1—C13—C14—C15	74.0 (2)
C1—C2—C3—C4	0.6 (3)	O2—C13—C14—C21	19.5 (3)
C2—C3—C4—C5	0.0 (3)	O1—C13—C14—C21	−161.95 (18)
C1—N1—C5—C4	0.7 (3)	C13—C14—C15—C16	−114.07 (19)
C1—N1—C5—C6	−179.70 (18)	C21—C14—C15—C16	122.80 (19)
C3—C4—C5—N1	−0.6 (3)	C13—C14—C15—C20	64.7 (2)
C3—C4—C5—C6	179.8 (2)	C21—C14—C15—C20	−58.5 (2)
N1—C5—C6—C7	171.0 (2)	C20—C15—C16—C17	−0.8 (3)
C4—C5—C6—C7	−9.4 (3)	C14—C15—C16—C17	178.00 (17)
C5—C6—C7—C8	179.33 (19)	C15—C16—C17—C18	0.5 (3)
C12—N2—C8—C9	−0.3 (3)	C16—C17—C18—C19	0.2 (3)
C12—N2—C8—C7	179.38 (18)	C17—C18—C19—C20	−0.5 (3)
C6—C7—C8—N2	173.9 (2)	C18—C19—C20—C15	0.3 (3)
C6—C7—C8—C9	−6.4 (4)	C16—C15—C20—C19	0.4 (3)
N2—C8—C9—C10	−0.2 (3)	C14—C15—C20—C19	−178.39 (18)
C7—C8—C9—C10	−179.9 (2)	C15—C14—C21—C22	−62.5 (2)
C8—C9—C10—C11	0.9 (4)	C13—C14—C21—C22	175.58 (17)
C9—C10—C11—C12	−0.9 (4)	C14—C21—C22—O3	−26.4 (3)
C8—N2—C12—C11	0.3 (3)	C14—C21—C22—O4	155.70 (18)
C10—C11—C12—N2	0.4 (4)		

### Hydrogen-bond geometry (Å, °)

**Table:**

*D*—H···*A*	*D*—H	H···*A*	*D*···*A*	*D*—H···*A*
O4—H4···N1	0.82	1.88	2.699 (2)	176
O1—H1···N2^i^	0.82	1.88	2.683 (2)	165
C2—H2···O2^ii^	0.93	2.56	3.472 (3)	164
C11—H11···O3^iii^	0.93	2.43	3.350 (3)	170
C3—H3···Cg1^iv^	0.93	2.86	3.650 (4)	143

Symmetry codes: (i) *x*, *y*+1, *z*−1; (ii) *x*−1, *y*, *z*+1; (iii) *x*+1, *y*−1, *z*; (iv) −*x*, −*y*+1, −*z*+1.

## References

[bb1] Bhogala, B. R., Basavoju, S. & Nangia, A. (2005). *Cryst. Growth Des.* **5**, 1683–1686.

[bb2] Bruker (1997). *SMART*, *SAINT* and *SADABS* Bruker AXS Inc., Madison, Wisconsin, USA.

[bb3] Fischer, A. & Profir, V. M. (2003*a*). *Acta Cryst.* E**59**, o319–o320.

[bb4] Fischer, A. & Profir, V. M. (2003*b*). *Acta Cryst.* E**59**, o485–o487.

[bb5] Fun, H.-K. & Kia, R. (2008). *Acta Cryst.* E**64**, m1116–m1117. 10.1107/S1600536808024306 PMC2960584 21201579

[bb6] Sheldrick, G. M. (2008). *Acta Cryst.* A**64**, 112–122. 10.1107/S0108767307043930 18156677

[bb7] Wang, Y.-T., Tang, G.-M., Zhang, Y.-C. & Wan, W.-Z. (2008). *Acta Cryst.* E**64**, o1753. 10.1107/S1600536808025567 PMC2960605 21201735

